# Supplementary material for: Sarcopenia in Neurological Patients: Standard Values for Temporal Muscle Thickness and Muscle Strength Evaluation
Source: J Clin Med. 2020 Apr 28;9(5):1272. doi: 10.3390/jcm9051272 (PMC7288067; doi:10.3390/jcm9051272)
Supplement: Supplementary file 1 [file jcm-09-01272-s001.pdf]

Supplementary Table 1: Clinical characteristics of the retrospective normal collective and the prospective patient cohort.

	Retrospective normal collective (n = 624)	Prospective patient cohort (n = 130)	p-value
**Sex**			
Female (n, %)	384 (62%)	73 (56%)	0.253
Male (n, %)	240 (38%)	57 (44%)	
**Age** (mean, SD, range; in years)	43 (17.8; 18 - 85)	47 (17.7; 18 - 79)	**0.004**
**TMT** (mean, SD, in mm)	8.6 (1.8)	7.8 (1.7)	**<0.001**
**Grip strength*** (mean, SD; in kg)	31.02 (10.93)	31.57 (12.05)	0.624
**BMI** (mean, SD)	27.6 (5.4)	25.8 (6.1)	**0.003**

* of the dominant hand Abbrevations: BMI: body mass index; SD: standard deviation; TMT: temporal muscle thickness.

Supplementary Table 2: Patient characteristics subdivided with regard to their disease entities.

	Neuro-Oncological (n = 44)	Cerebro-Vascular (n = 28)	CNS-Demyelinating (n = 15)	Psychiatric (n = 12)	Others (n = 31)
**Sex**					
Female	19(43%)	10 (36%)	5 (33%)	4 (33%)	19 (61%)
Male	25(57%)	18 (64%)	10 (67%)	8 (67%)	12 (39%)
**Age** (mean, SD, range; in years)	46 (18.2; 18-78)	55 (17.3; 18-79)	35 (10.4; 18-52)	38 (12.3; 20-65)	51 (17.1; 18-78)
**TMT** (mean, SD; in mm)	7.4 (1.9)	7.6 (1.5)	7.2 (1.4)	8.4 (1.5)	8.2 (1.8)
**Grip strength *** (mean, SD; in kg)	31.5 (12.6)	31.8 (9.4)	34.8 (14.9)	34.4 (11.7)	33.5 (11.7)
**BMI** (mean, SD)	25.2 (5.3)	26.8 (6.0)	24 (4.6)	24 (5.7)	27.3 (7.7)
**Temporal wasting** (present; n, %)	12 (27%)	7 (25%)	1 (7%)	5 (42%)	8 (26%)
**Unintended weight loss** (n, %)	18 (41)	6 (21)	3 (20)	6 (50)	4 (13)
**NANO scale** (mean, range)	2 (2.48; 0 – 9)	1.7 (1.12; 0 - 5)	2.7 (2.52; 0 – 10)	1.2 (1.80; 0 – 5)	1.1 (1.93; 0 – 9)

* dominant hand Abbrevations: CNS: central nervous system; BMI: body mass index; NANO: Neurological Assessment of Neuro-Oncology; SD: standard deviation; TMT: temporal muscle thickness.
